# Efficacy and safety of fezolinetant, a neurokinin-3 antagonist, in treating vasomotor symptoms in postmenopausal women: A systematic review and meta-analysis

**DOI:** 10.1097/MD.0000000000036592

**Published:** 2023-12-15

**Authors:** Ummi Aiman Rahman, Talha Bin Kashif, Muhammad Usman, Maham Rana, Muhammad Hasanain, Muhammad Umair Anjum, Huzaifa Ahmad Cheema, Huda Jaffar, Pratik Bhattarai

**Affiliations:** a Department of Medicine, King Edward Medical University, Lahore, Pakistan; b Department of Medicine, Dow Medical College, Dow University of Health Sciences, Karachi, Pakistan; c Department of Medicine, Manipal College of Medical Sciences, Pokhara, Nepal.

**Keywords:** fezolinetant, hot flashes, menopause, neurokinin-3 receptor antagonist, quality of life, vasomotor symptoms, VMS

## Abstract

**Background:**

Menopause causes a variety of symptoms such as hot flashes and night sweats. While menopausal hormonal therapy has been used for managing postmenopausal vasomotor symptoms (VMS) for quite a while, it has a considerably poor safety profile.

**Objective:**

To review and analyze existing data to evaluate the efficacy of the neurokinin-3 antagonist, fezolinetant, in treating postmenopausal VMS and to assess its safety profile.

**Methods:**

A thorough literature search was performed on PubMed, Cochrane Library, and Google Scholar in compliance with Preferred Reporting Items for Systematic Reviews and Meta-Analysis 2020, to find publications on the efficacy of fezolinetant for postmenopausal VMS. Changes in the frequency and severity scores of moderate/severe VMS and changes in the Hot Flash-Related Daily Interference Scale (HFRDIS), Greene Climacteric Scale (GCS), and Menopause-Specific Quality of Life (MENQoL) were the efficacy outcomes. Adverse events, drug-related treatment-emergent adverse effects (TEAEs), drug-related dropouts, hepatotoxicity, endometrial hyperplasia or tumor, and uterine bleeding were all safety outcomes. We used Review Manager 5.4 for pooling risk ratios (RRs) and mean differences (MDs) for dichotomous and continuous outcomes, respectively. A *P* value of < .05 was considered significant.

**Results:**

There was a significant reduction in mean daily VMS frequency at weeks 4 and 12 (MD, −2.36; 95% confidence interval [CI], −2.85 to −1.87; *P* < .00001, for week 12) and also a significant decrease in VMS severity scores in the treatment group. Furthermore, improvements in MENQoL, HFRDIS, and GCS scores were observed. There was no significant difference in adverse events while drug-related TEAEs (RR, 1.21; 95% CI, 0.90–1.63; *P* = .21) showed a slight increase with fezolinetant. Drug-related dropouts were again similar across the 2 groups. Uterine bleeding had a lower incidence while endometrial events and hepatotoxicity showed a statistically insignificant, increasing trend in the fezolinetant group.

**Discussion and implications:**

Fezolinetant can be a treatment option for postmenopausal VMS but warns of a risk increase in endometrial hyperplasia or tumors. The heterogeneity in the data being analyzed, short follow-up period, and small sample size in most of the included randomized controlled trials were the greatest limitations, which must be considered in further research and safety profile exploration.

## 1. Introduction

Menopause is a challenging physiological process that marks the end of a woman’s reproductive life. There is a progressive decline in the production of reproductive hormones (estrogen and progesterone) by the ovaries. This transition results in a variety of symptoms, of which hot flashes and night sweats, emotional disturbances, urogenital complaints, and disrupted sleep are only a few. These add up to the social adjustments an aging woman must make.^[1]^ While menopause is often natural, procedures such as hysterectomy or bilateral oophorectomy result in surgical menopause, where hormonal variations are more abrupt and symptoms more pronounced.^[2]^

Despite having a troublesome impact on the overall quality of life (QoL) of perimenopausal and postmenopausal women, vasomotor symptoms (VMS), which include hot flashes and night sweats, remain understudied, with only a few therapy options available.^[3,4]^ The available treatments are not widely accessible (cognitive behavioral therapy^[5]^), are limitedly efficacious (e.g., selective serotonin reuptake inhibitors^[6]^), cause treatment-related adverse effects, are contraindicated (menopausal hormone therapy [MHT]) in women with personal or family history positive for breast cancer,^[7]^ or are avoided in general due to long-term therapy risks, such as in case of estrogen-progesterone combined hormonal therapy (HT),^[8]^ especially after Women’s Health Initiative showed an increased risk of coronary heart disease and breast cancer.^[9]^

A few randomized controlled trials (RCTs) were conducted recently to test the efficacy of neurokinin-3 (NK-3) antagonist, fezolinetant, for treating postmenopausal hot flashes in an effort to discover a novel, effective, and targeted nonhormonal medication for VMS.^[10–15]^ Evidence suggests a link between the expression of the neuropeptide neurokinin B (NKB) by kisspeptin/neurokinin B/dynorphin (KNDy) neurons in the hypothalamus and the incidence of hot flashes via the regulation of the autonomic thermoregulatory pathway through the median preoptic nucleus. It has been proposed that the blockade of NK-3 receptors with NK-3 antagonists can help attenuate VMS without needing the administration of estrogen. The main idea is to limit excessive NKB-induced NK-3 stimulation in the absence of sex hormones to control the thermoregulatory system.^[16]^ The use of fezolinetant for the treatment of VMS was authorized by the US Food and Drug Administration in May 2023, following the conclusion of 2 phase III RCTs.^[17]^

Since 2019, several trials that evaluated the efficacy of fezolinetant for treating hot flashes and night sweats, and its effect on postmenopausal women’s QoL while also assessing its safety, have published their findings.^[10–15]^ In this article, we aim to systematically review and thoroughly analyze the published results by performing a meta-analysis of the RCTs to pool the outcomes into one study. This, to our knowledge, is the first meta-analysis on this subject, which will help to demonstrate how well the NK-3 antagonist works in lowering VMS severity scores and reducing the VMS frequency and will also establish the drug’s overall safety profile, advancing the quest for the much-awaited wonder drug a step closer.

## 2. Methods

This systematic review and meta-analysis was carried out according to the guidelines available in the Cochrane Handbook for Systematic Reviews of Interventions^[18]^ and reported according to the Preferred Reporting Items for Systematic Reviews and Meta-Analysis (PRISMA) 2020 guidelines.^[19]^ Since it is a systematic review and meta-analysis of already published original studies (RCTs) and involves pooling of the data with no direct involvement of the patients, ethical approval was not required, and hence, none taken.

### 2.1. Data search

An electronic search from PubMed, Cochrane Library, and Google Scholar was performed by 2 independent authors (Rahman and Kashif) from inception up to June 30, 2023 to identify studies conducted on human subjects that assessed the efficacy of NK-3 receptor inhibition via fezolinetant, on postmenopausal symptoms such as hot flashes and night sweats. The results were not limited by any language or any filter. The keywords used for the literature search included, “Fezolinetant,” “ESN364,” “NK3 Receptor Antagonist,” “Menopause,” “Menopausal Symptoms,” “Flashes,” “Vasomotor Symptoms,” and “VMS.” We also screened reference lists of included studies and similar systematic reviews to identify further relevant studies. The detailed search strategy is included in the supplementary data (see Table S1, Supplemental Digital Content, http://links.lww.com/MD/L36).

### 2.2. Eligibility criteria

We included only RCTs in our meta-analysis. The inclusion criteria for our meta-analysis were: patient population, adult postmenopausal females experiencing VMS; intervention and control, trials that involved different dosage regimens of fezolinetant administered to patients for VMS versus placebo.

The exclusion criteria were as follows: all study designs other than RCTs, such as quasirandomized trials and other observational studies; studies not reported in the English language; studies reporting in vivo or in vitro experiments, or those having nonhuman population; and studies not reporting the relevant outcomes.

### 2.3. Study selection and data extraction

The studies yielded by our search strategy were imported into Mendeley Desktop 1.19.8 (Mendeley Ltd, Amsterdam, The Netherlands) and duplicates were removed. Two authors (Rahman and Kashif) independently screened the titles and abstracts of all the retrieved articles and removed those not fulfilling the eligibility criteria. The full texts of the remaining articles were reviewed against the eligibility criteria. Conflicts or disagreements were discussed and resolved by a third author (Hasanain).

Data extraction was undertaken independently by 2 researchers (Rahman and Kashif). In case of any disagreements, the data were discussed with a third researcher (Hasanain). Data regarding study characteristics (including authors, study design, and outcomes), patient population (including the number of participants, age, and baseline characteristics), and intervention (dosage regimens) were extracted. Values for graphically represented data were extrapolated from the graphs using Web Plot Digitizer^[20]^ whenever not reported in the text.

### 2.4. Outcomes

The primary outcomes were changes in the “frequency” of moderate/severe VMS from baseline and changes in the “severity” of moderate/severe VMS from baseline assessed at 4 and 12 weeks. Secondary outcomes included changes in the Hot Flash-Related Daily Interference Scale (HFRDIS), Greene Climacteric Scale (GCS), and Menopause-Specific Quality of Life (MENQoL) from baseline, number of patients reporting any adverse event, drug-related treatment-emergent adverse effects (TEAEs), hepatotoxicity, endometrium-related adverse effects, and dropouts due to drug-related TEAEs. The MENQoL serves as an effective tool for evaluating the symptoms experienced by women during menopause. The HFRDIS is a 10-item questionnaire that evaluates the effects of hot flashes on a woman’s daily life. The GCS is a 21-item scale used to assess symptoms related to the climacteric phase.

### 2.5. Risk of bias assessment

The revised Cochrane Risk of Bias Tool (RoB 2.0) for RCTs^[21]^ was used to evaluate the risk of bias in the studies included in our analysis. RoB 2.0 assesses bias in 5 domains: bias resulting from the randomization process; bias due to deviations from intended interventions; bias due to missing outcome data; bias in the measurement of the outcome; and bias in the selection of the reported results. Each study was analyzed based on each of these 5 domains and assigned as having high, low, or some concerns of bias.

### 2.6. Statistical analysis

We conducted the statistical analysis using Review Manager (RevMan, Version 5.4; The Cochrane Collaboration, Copenhagen, Denmark). Risk ratios (RRs) and mean differences (MDs) for dichotomous and continuous outcomes, respectively, with 95% confidence intervals (CIs) were pooled using random-effects model. A *P* value of < .05 was considered significant. RevMan calculator was used, in case the standard deviation (SD) was not given for a continuous outcome, which helped to convert CI or standard error (SE) into SD. The χ^2^ test and Higgins I2 statistic were used to assess heterogeneity across studies. Publication bias could not be assessed due to the limited number of RCTs available for analysis. Subgroup analyses were done based on dosage regimens.

## 3. Results

### 3.1. Literature search results

**Figure 1.**
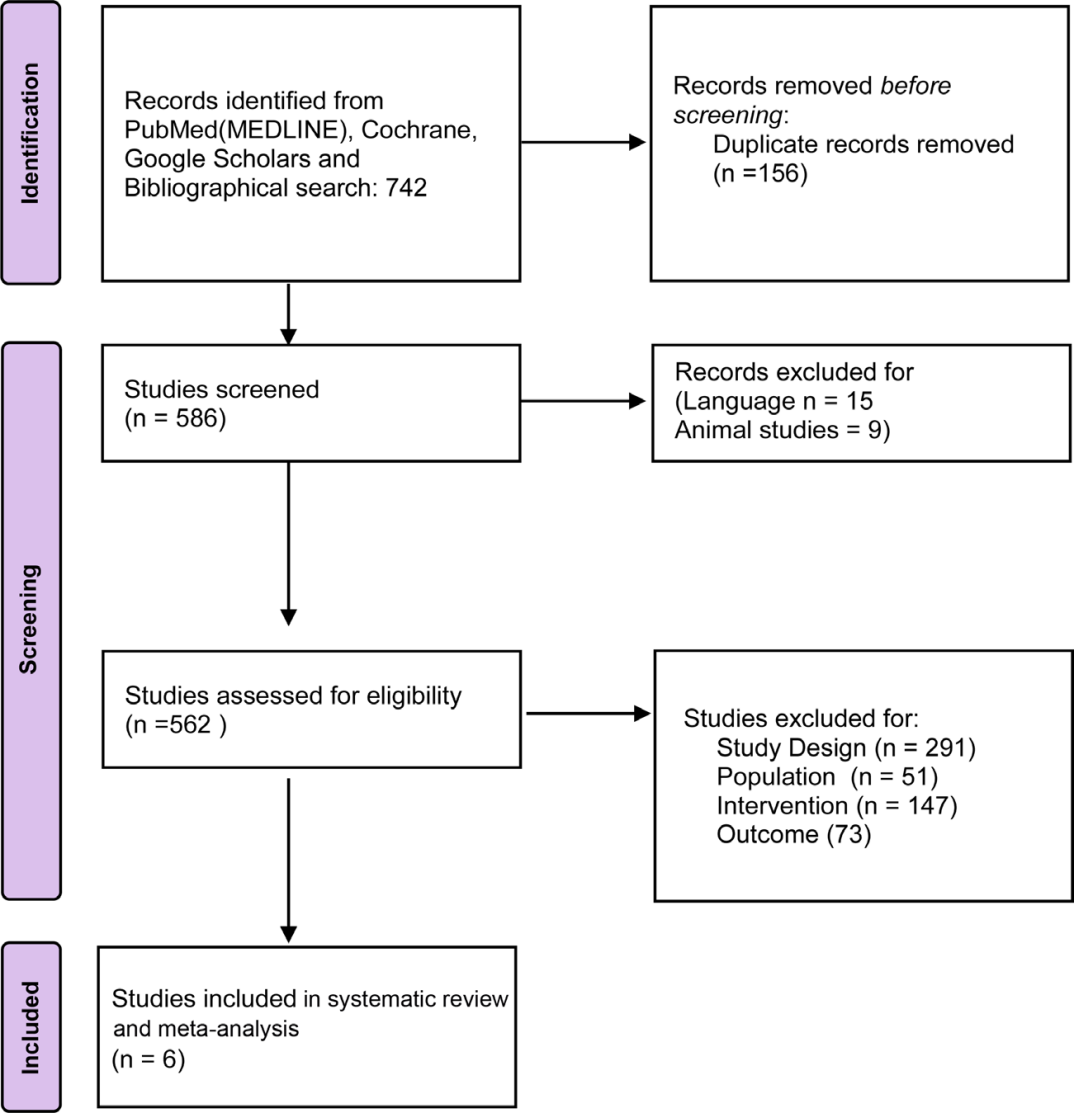
PRISMA 2020 flow chart for the selection of eligible studies. PRISMA *=* Preferred Reporting Items for Systematic Reviews and Meta-analyses.

The search strategy retrieved 742 articles. After removing duplicate studies and screening full-text articles, we included a total of 6 full-text papers (5 RCTs) in our systematic review and meta-analysis. The literature screening process is summarized in the PRISMA flowchart in Figure 1.

### 3.2. Study characteristics

We included 5 RCTs in our systematic review and meta-analysis. The studies were conducted in different regions of the world: the United States, Canada, the United Kingdom, Czech Republic, Poland, Belgian centers, Hungary, and Spain. The median age ranged from 53.5 to 54.6 years, and the follow-up period was 2 to 3 weeks. The study characteristics of the included studies are presented in Table 1.

**Table 1:** Characteristics of included studies.

Study ID	Study design	Trial setting	Trial duration	Dosage, frequency of dosing		Follow-up period (wk)	Sample size (intervention vs control)	Age of participants, mean years (SD)	Hysterectomy (n)	Baseline VMS mean frequency/d	Moderate/severe VMS score, mean daily
				FEZ	Control						
Depypere et al,^[10]^ EudraCT (2015-002578-20)	RCT	8 Belgian centers	12 wk	90 mg BID for 12 wk	Placebo BID	2–3 wk	FEZ 90 mg BID = 43 PBO = 44	53.5 (4.14)	NR	FEZ = 80.7 episodes/wk (95% CI, 70.6–90.8) PBO = 72.0 episodes/wk (95% CI, 63.9–80.1)	FEZ = 27.8 (95% CI, 23.7–32.0) PBO = 24.9 (95% CI, 21.7–28.1)
Fraser et al,^[11]^ Santoro et al,^[12]^ NCT03192176	RCT	51 sites in the United States	12 wk	90 mg BID 30 mg QD	PBO BID	3 wk	FEZ 90 mg BID = 44 FEZ 30 mg QD = 45 PBO = 44	54.6	NR	NR	NR
Johnson et al,^[14]^ NCT04003142	RCT	146 sites across the United States, Canada, Czechia, Latvia, Poland, Spain, and United Kingdom	52 wk (12 wk blinding + 40 wk extension period)	30 mg OD 45 mg OD	PBO OD	3 wk	FEZ 30 mg = 166 FEZ 45 mg = 167 PBO = 167	54.3 (5.0)	FEZ 30 mg = 53/166 FEZ 45 mg = 56/167 PBO = 51/167	FEZ 30 mg = 11.23 (SD, 4.88) FEZ 45 mg = 11.79 (SD, 8.26) PBO = 11.59 (SD, 5.02)	FEZ 30 mg = 2.44 (SD, 0.33) FEZ 45 mg = 2.41 (SD, 0.34) PBO = 2.41 (SD, 0.32)
Lederman et al,^[13]^ NCT04003155	RCT	97 facilities across the United States, Canada, Czech Republic, Hungary, Poland, Spain, and the United Kingdom	52 wk (12 wk blinding + 40 wk extension period)	30 mg 45 mg (once daily)	PBO (once daily, 12 wk)	3 wk	FEZ 30 mg = 173 FEZ 45 mg = 174 PBO = 175	FEZ 30 mg = 54.2 FEZ 45 mg = 54.2 PBO = 54.7	FEZ 30 mg = 61/174 FEZ 45 mg = 56/173 PBO = 51/175	FEZ 30 mg = 10.7 (SD, 4.7) FEZ 45 mg = 10.4 (SD, 3.9) PBO = 10.5 (SD, 3.8)	NR
Neal-Perry et al,^[15]^ NCT04003389	RCT	NR	52 wk	30 mg 45 mg (once daily)	PBO	3 wk	FEZ 30 mg = 611 FEZ 45 mg = 609 PBO = 610	FEZ 30 mg = 54.7 FEZ 45 mg = 54.7 PBO = 54.9	FEZ 30 mg = 100/611 FEZ 45 mg = 114/609 PBO = /610	NR	NR

CI = confidence interval, FEZ = fezolinetant, PBO = placebo, RCT = randomized control trial, SD = standard deviation, VMS = vasomotor symptoms.

### 3.3. Quality assessment

**Figure 2.**
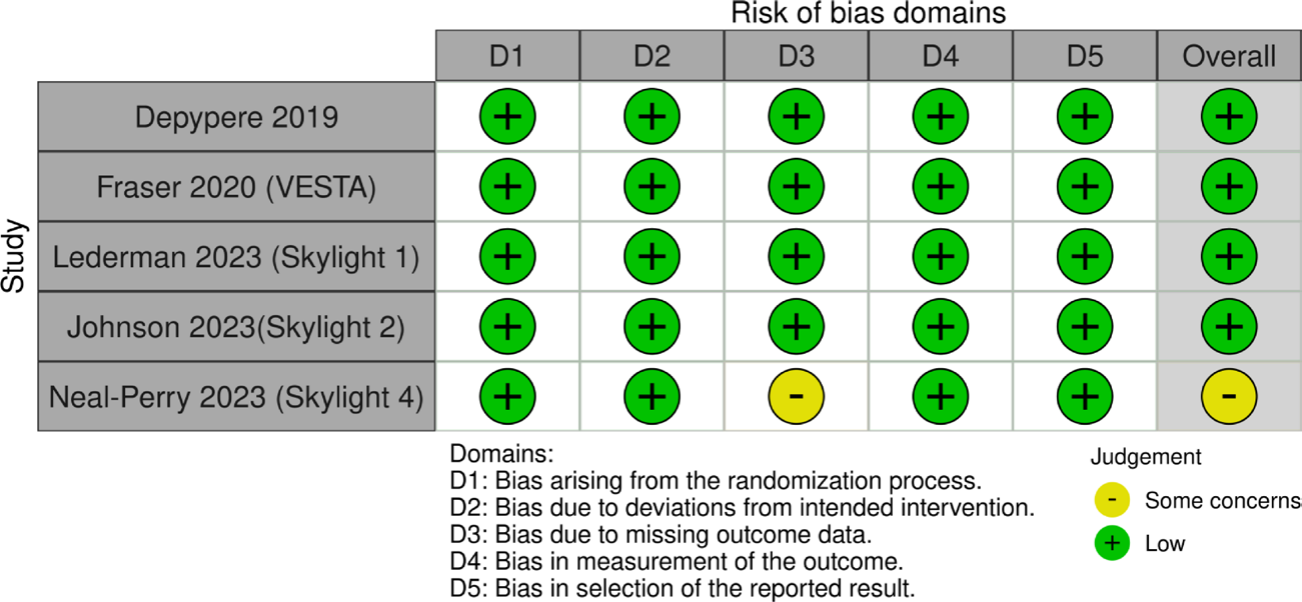
Assessment of study-by-study risk of bias via Cochrane Risk of Bias 2 (RoB 2) tool.

The risk of bias of included studies was assessed using RoB 2.0. All the studies showed a low risk of bias except one with some concerns (due to concerns in the domain of reporting missing outcome data). Overall, the studies were of high quality, and the risk of bias was low, as shown in Figure 2.

### 3.4. Efficacy outcomes

#### 3.4.1. Changes in daily frequency of moderate/severe VMS.

**Figure 3.**
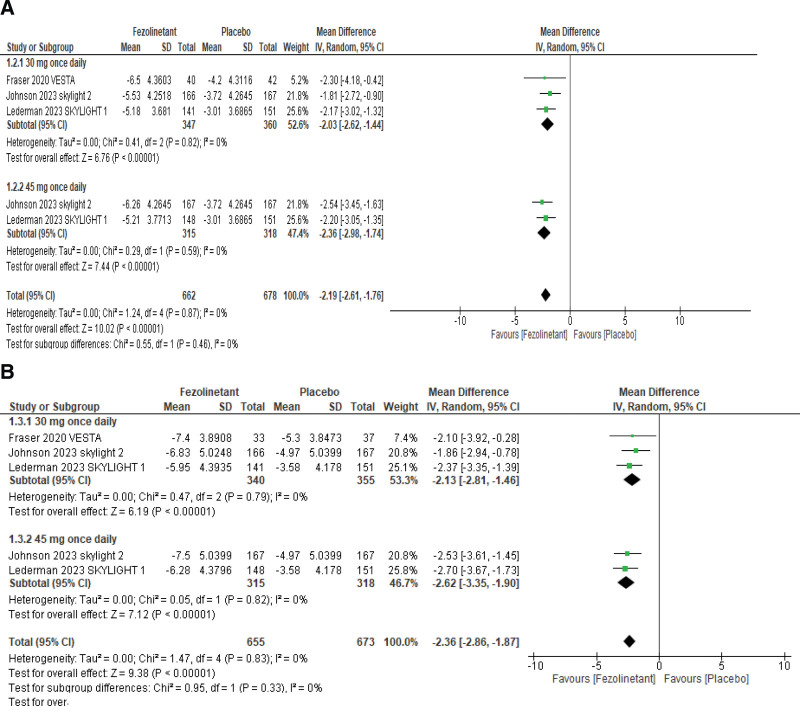
Forest plot for change from baseline in daily frequency of moderate/severe VMS at (A) week 4 and (B) week 12.

Three studies reported changes in the daily frequency of moderate/severe VMS at weeks 4 and 12. The overall pooled results (Fig. 3A) at week 4 showed that fezolinetant is associated with a significant reduction in mean daily VMS frequency (MD, −2.19; 95% CI, −2.61 to −1.76; *P* < .00001; I2 = 0%). A subgroup analysis was performed based on administered doses of fezolinetant. Three studies^[11,13,14]^ studied the outcome with the usage of 30 mg QD and 2 studies^[13,14]^ used 45 mg QD. Analysis of these subgroups showed that both doses cause statistically significant improvements in VMS frequency compared to placebo.

Pooled analysis at week 12 (Fig. 3B) demonstrated that all treatment groups exhibited a decrease in the frequency of moderate/severe VMS (MD, −2.36; 95% CI, −2.85 to −1.87; *P* < .00001; I2 = 0%). Studies were also analyzed within different subgroups according to the doses administered. Three studies^[11,13,14]^ studied the outcome with the usage of 30 mg QD and 2 studies^[13,14]^ used 45 mg QD. Results of subgroup analyses showed that both fezolinetant doses met statistical significance in reducing VMS frequency at week 12.

A fourth study, Depypere et al,^[10]^ had also reported a change in the moderate/severe VMS frequency from baseline; however, the data could not be analyzed since it reported VMS frequency on a weekly basis, unlike others (which reported the moderate/severe VMS daily frequency). Nonetheless, the study reported a significant reduction of moderate/severe VMS frequency with fezolinetant at both weeks 4 and 12.

#### 3.4.2. Changes in daily severity of moderate/severe VMS.

**Figure 4.**
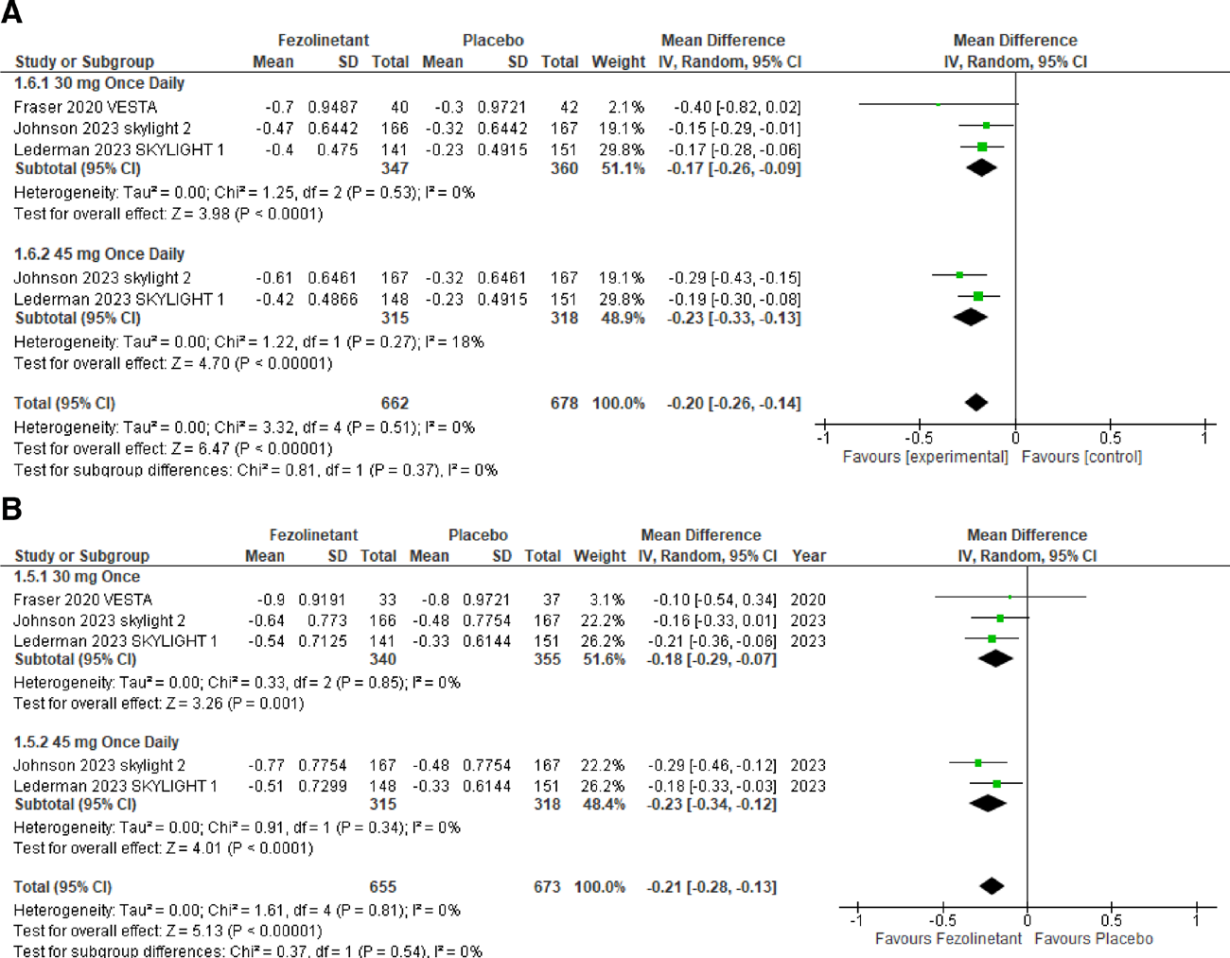
Forest plot for change from baseline in daily severity of moderate/ severe VMS at (A) week 4 and (B) week 12. VMS = vasomotor symptoms.

Changes in daily severity of moderate/severe VMS scores were analyzed at weeks 4 and 12 (Fig. 4A, B).

This outcome was reported at week 4 by 3 studies^[11,13,14]^ including 1340 patients. Pooled analysis of all these studies at different dosages showed significant results favoring treatment with fezolinetant over placebo showing a decrease in score in the treatment group (MD, 0.20; 95% CI, −0.26 to −0.14; *P* < 00001; I2 = 0%). A fourth study^[10]^ had also reported this outcome but could not be included in the analysis since there was heterogeneity in the way severity scores were calculated compared to the other 3 studies. Regardless, the study also showed a significant reduction in VMS severity scores for fezolinetant, at both weeks 4 and 12.

The outcome was stratified according to the doses used. Three studies^[11,13,14]^ studied the outcome with the usage of 30 mg QD and 2 studies^[13,14]^ used 45 mg QD. Analysis of these subgroups showed a decrease in both 30mg QD (MD, 0.17; 95% CI, −0.26 to −0.09) and 45 mg QD subgroups (MD, 0.23; 95% CI, −0.33 to −0.13).

At week 12, 3 studies^[11,13,14]^ reported daily severity of moderate/severe VMS. Pooled analysis of these studies with 1328 patients showed significant results favoring treatment with fezolinetant over placebo, showing a decrease in the score in the treatment group (MD, 0.21; 95% CI, −0.28 to −0.13; *P* < .00001; I2 = 0%).

Studies were also analyzed within different subgroups according to the doses administered. Analysis of the 3 studies^[11,13,14]^ using 30 mg QD showed a decrease in the scores (MD, 0.18; 95% CI, −0.29 to −0.07). Similarly, analysis of studies using 45 mg QD fezolinetant^[13,14]^ also showed a decrease in mean daily severity score (MD, 0.23; 95% CI, −0.34 to −0.12).

#### 3.4.3. Menopause-Specific Quality of Life (MENQoL).

**Figure 5.**
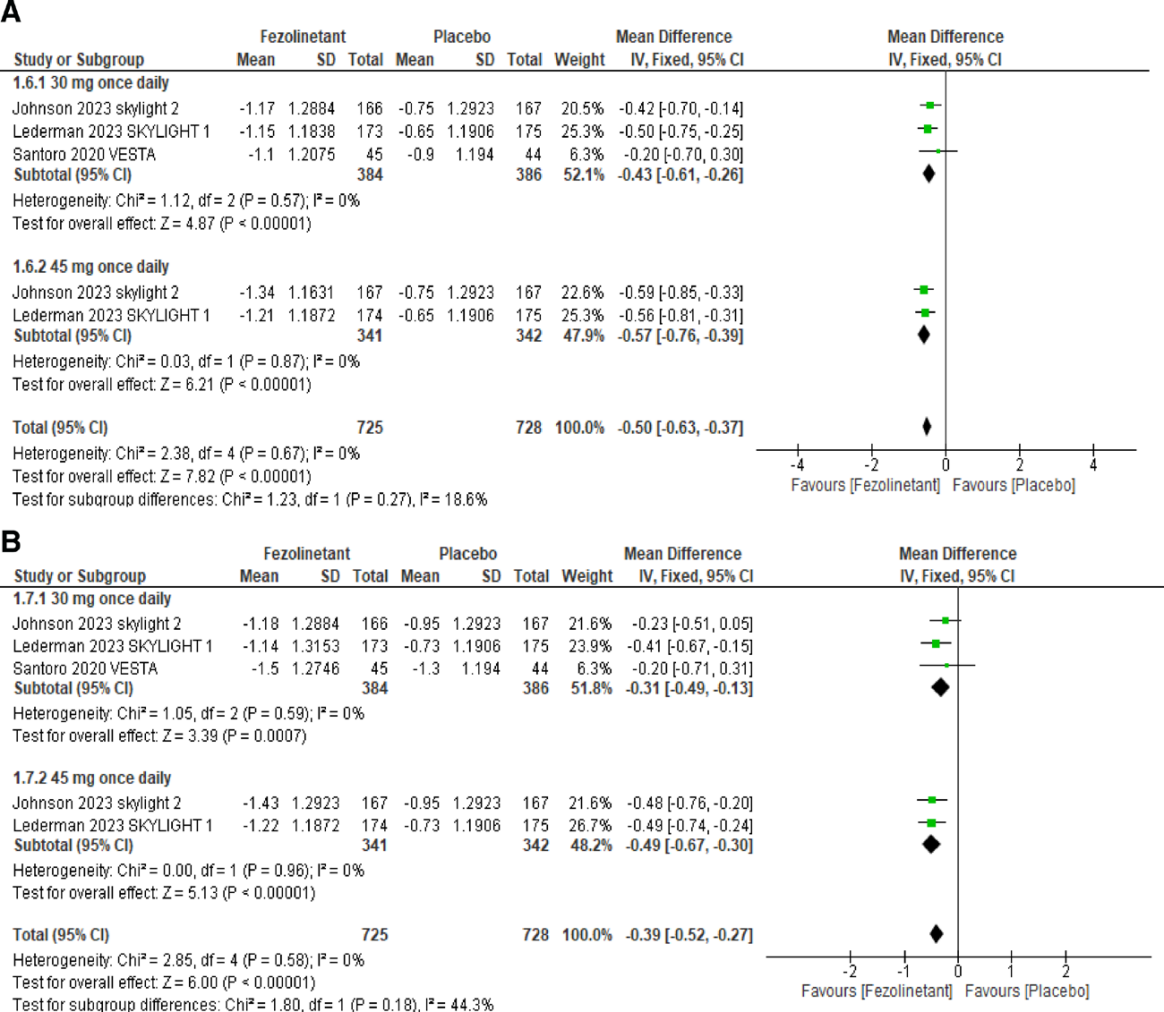
Forest plot for changes from baseline in MENQoL scores at (A) week 4 and (B) week 12. MENQoL = Menopause-Specific Quality of Life.

Improvements in MENQoL were observed by 3 studies at weeks 4 and 12 (Fig. 5A, B). The overall pooled results at week 4 showed that fezolinetant is associated with a significant improvement in MENQoL total score (MD, −0.50; 95% CI, −0.63 to −0.37; *P* < .00001; I2 = 0%). A subgroup analysis was performed based on administered doses of fezolinetant. Three studies^[12–14]^ assessed the outcome with the usage of 30 mg QD, and 2 studies^[13,14]^ used 45 mg QD. Analysis of these subgroups showed that each fezolinetant dose substantially improved QoL (MENQoL).

Pooled analysis at week 12 demonstrated that all treatment groups exhibited a significant improvement in MENQoL total score (MD, −0.39; 95% CI, −0.52 to −0.27; *P* < .00001; I2 = 0%). Additional subgroup analyses were performed for different subgroups according to the doses administered. Three studies^[12–14]^ assessed the outcome with the usage of 30 mg QD and 2 studies^[13,14]^ used 45 mg QD. Results of subgroup analyses showed that both doses of fezolinetant met statistical significance in improving the MENQoL total score at week 12.

#### 3.4.4. Hot Flash-Related Daily Interference Scale (HFRDIS).

**Figure 6.**
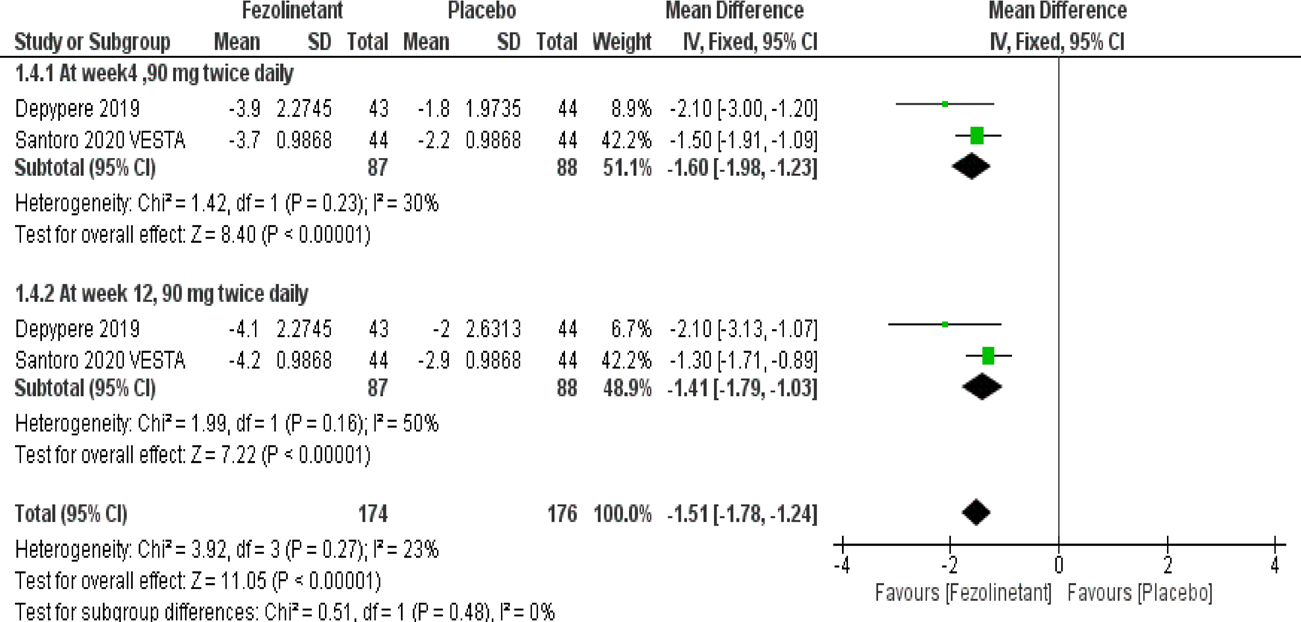
Forest plot for changes from baseline in HFRDIS scores. HFRDIS = Hot Flash-Related Daily Interference Scale.

HFRDIS was investigated by 2 studies.^[10,12]^ Pooled analysis (Fig. 6) showed that fezolinetant led to substantial improvement in HFRDIS (MD, −1.51; 95% CI, −1.78 to −1.24; *P* < .00001; I2 = 23%). Consistent findings were seen in stratified analysis in both 90 mg BID groups at week 4 (MD, −1.60; 95% CI, −1.98 to −1.23; *P* < .00001; I2 = 30%) and week 12 (MD, −1.41; 95% CI, −1.79 to −1.03; *P* < .00001; I2 = 50%).

#### 3.4.5. Greene Climacteric Scale (GCS).

**Figure 7.**
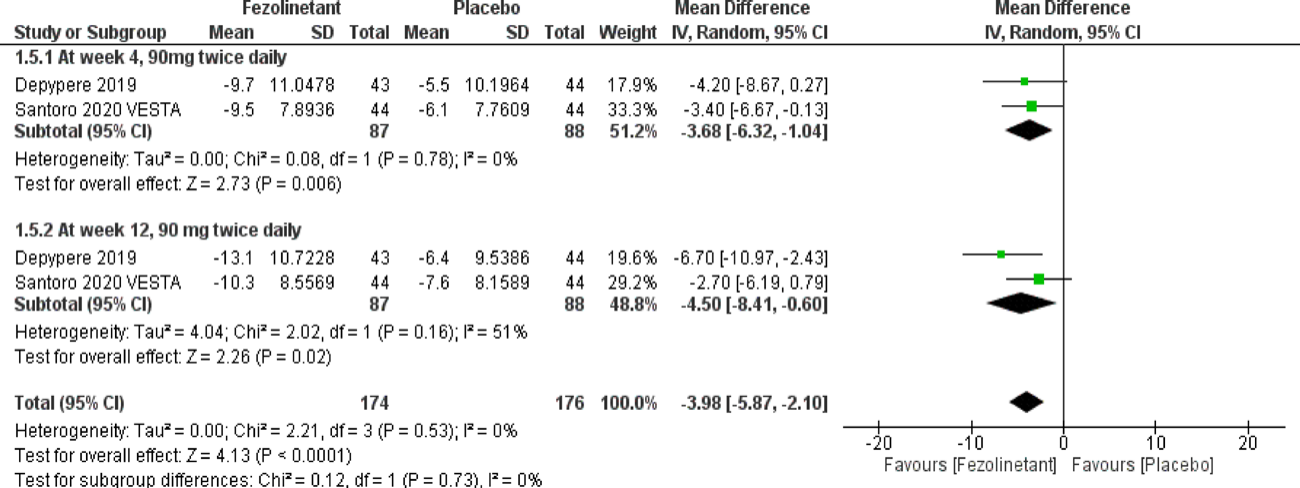
Forest plot for changes from baseline in GCS scores. GCS = Greene Climacteric Scale.

GCS was examined in 2 studies.^[10,12]^ The overall pooled results (Fig. 7) demonstrated that fezolinetant is associated with a significant improvement in GCS (MD, −3.98; 95% CI, −5.87 to −2.10; *P* < .00001; I2 = 0%). These consistent results also applied to the stratified analysis of the 90 mg BID group at week 4 (MD, −3.68; 95% CI, −6.32 to −1.04; *P* = .006; I2 = 0%) and week 12 (MD, −4.50; 95% CI, −8.41 to −0.60; *P* = .02; I2 = 51%).

### 3.5. Safety outcomes

#### 3.5.1. Any adverse events.

**Figure 8.**
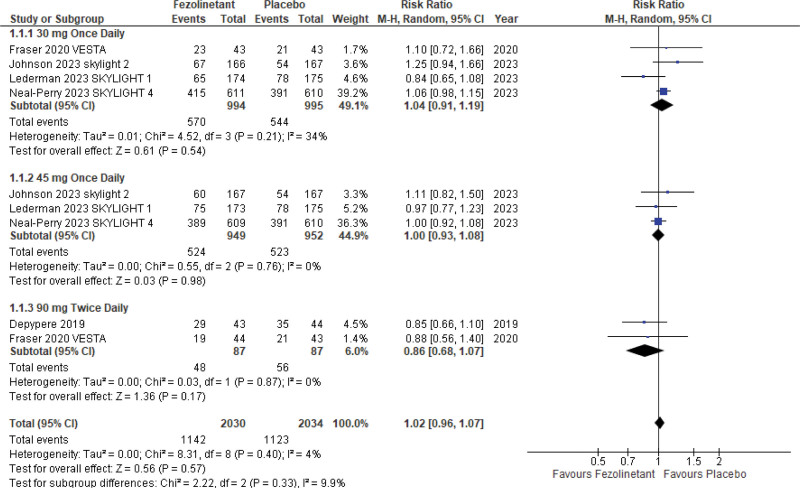
Forest plot for any adverse event.

Five studies^[10,11,13–15]^ with 4064 patients reported all adverse events throughout the treatment duration. These studies used different dosages, including 30 mg QD, 45 mg QD, and 90 mg BID. Pooled analysis (Fig. 8) of all these dosages showed no significant difference between fezolinetant and placebo (RR, 1.02; 95% CI, 0.96–1.07; *P* = .57; I2 = 4%).

Subgroup analysis was performed on these studies as they were stratified according to different doses. Of these, 4 studies^[11,13–15]^ reported these events with the usage of 30 mg QD while 3 of these studies^[13–15]^ also reported adverse events with 45 mg QD dosage and 2 studies^[10,11]^ reported adverse events with 90 mg BID. The RR value determined for 30 mg QD was 1.04 (95% CI, 0.91–1.19), 45 mg QD was 1.00 (95% CI, 0.93–1.08), and 90 mg BID was 0.86 (95% CI, 0.68–1.07).

#### 3.5.2. Drug-related TEAEs.

**Figure 9.**
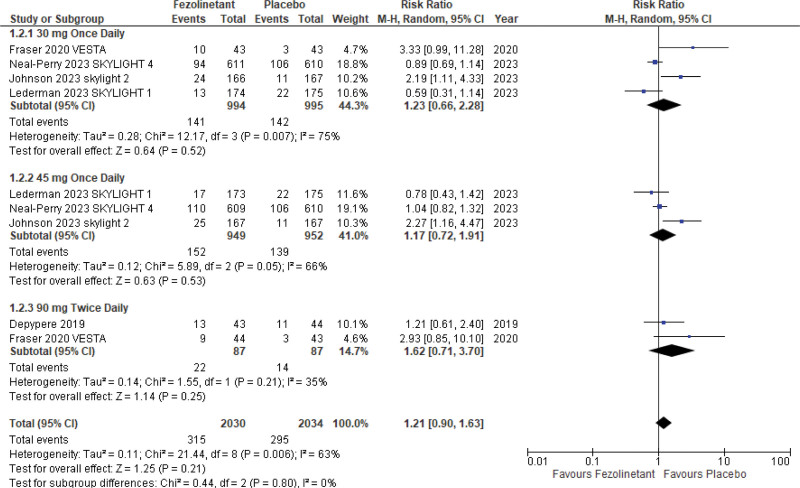
Forest plot for drug-related TEAEs. TEAEs* = *treatment-emergent adverse effects.

Drug-related treatment-emergent adverse events were reported by 5 studies^[10,11,13–15]^ (4064 patients). The risk of drug-related adverse events was slightly increased in the fezolinetant group. However, the results were not statistically significant (RR, 1.21; 95% CI, 0.90–1.63; *P* = .21; I2 = 63%) (Fig. 9).

Consistent findings were seen in stratified analysis in 30 mg QD (RR, 1.23; 95% CI, 0.66–2.28), 45 mg QD (RR, 1.17; 95% CI, 0.72–1.91), and 90 mg BID (RR, 1.62; 95% CI, 0.71–3.70).

#### 3.5.3. Dropouts/discontinuation due to drug-related TEAEs.

**Figure 10.**
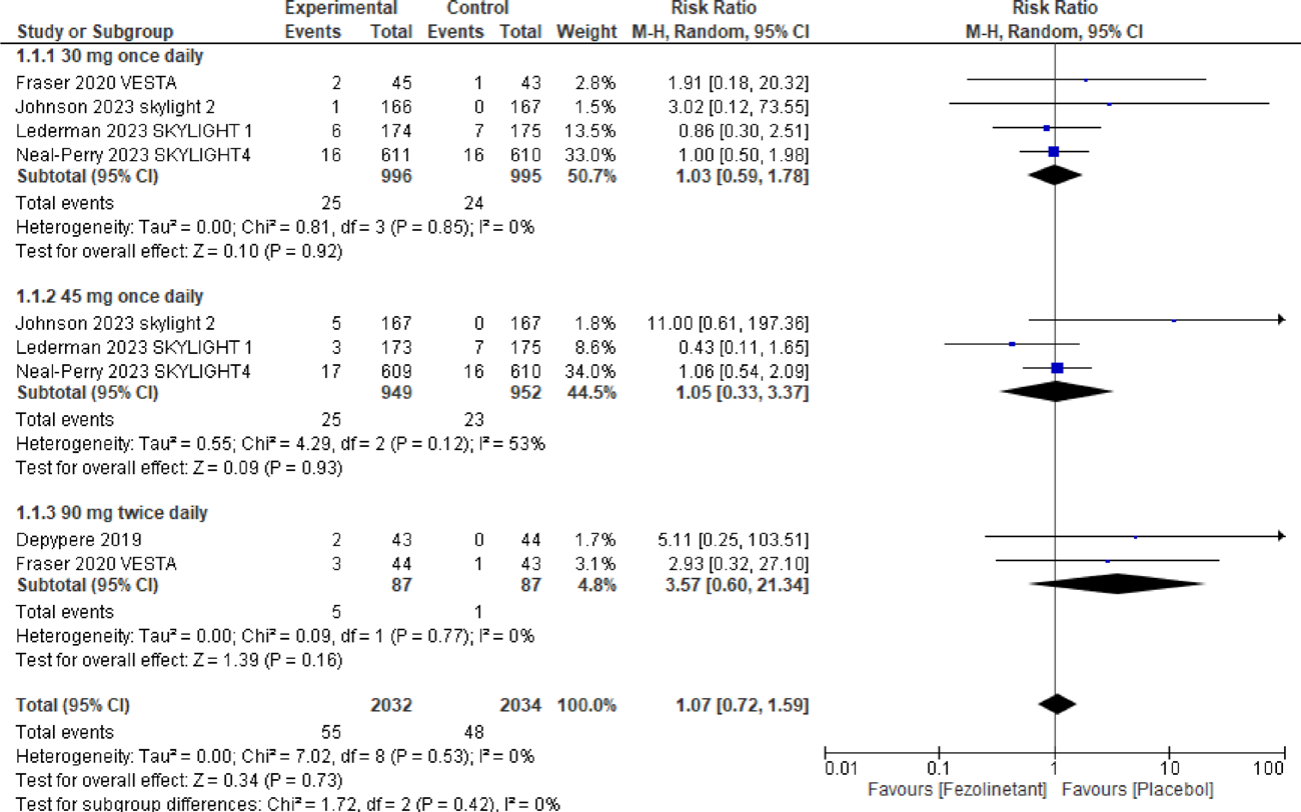
Forest plot for dropouts/discontinuation due to drug-related TEAEs. TEAEs = treatment-emergent adverse effects.

Dropouts due to drug-related TEAEs were reported by 5 studies^[10,11,13–15]^ (4064 patients). The overall pooled results (Fig. 10) showed that there was no statistically significant difference between fezolinetant and placebo (RR, 1.07; 95% CI, 0.72–1.59; *P* = .73; I2 = 0%).

Consistent findings were seen in stratified analysis in 30 mg QD (RR, 1.03; 95% CI, 0.59–1.78) and 45 mg QD (RR, 1.05; 95% CI, 0.33–3.37) as well as 90 mg BID (RR, 3.57; 95% CI, 0.60–21.34).

#### 3.5.4. Hepatotoxicity.

**Figure 11.**
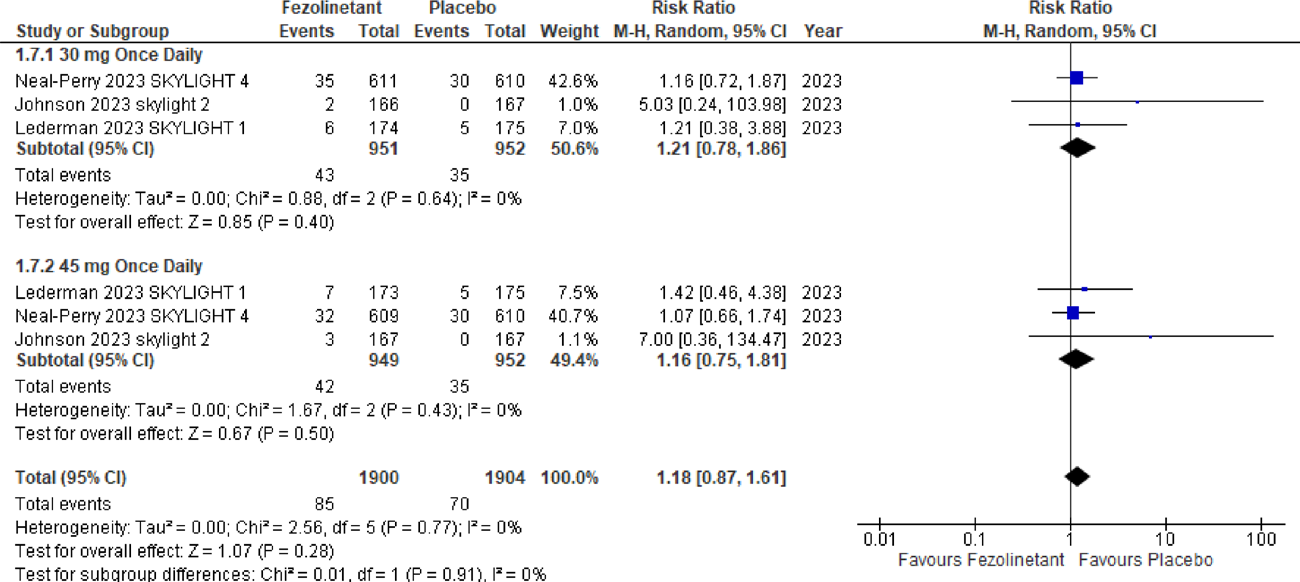
Forest plot for hepatotoxicity.

Three studies^[13–15]^ included data on hepatotoxicity (3804 patients) using increased liver function tests (LFTs) as a marker. Pooled analysis (Fig. 11) revealed no significant difference between the risk of LFTs elevation in both groups (RR, 1.18; 95% CI, 0.87–1.61; *P* = .28; I2 = 0%).

In an analysis stratified by different doses, similar effects were observed with treatment with 30 mg QD (RR, 1.21; 95% CI, 0.78–1.86) as well as 45 mg QD (RR, 1.16; 95% CI, 0.75–1.61).

#### 3.5.5. Endometrial hyperplasia/tumor.

**Figure 12.**
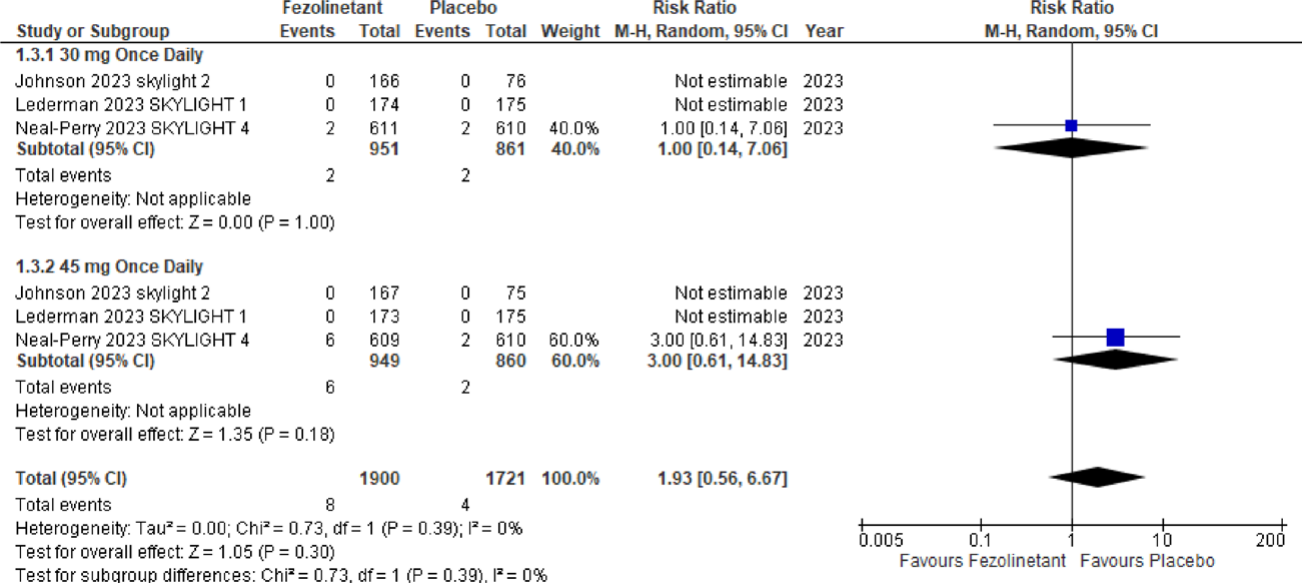
Forest plot for endometrial hyperplasia and tumors.

Three studies^[13–15]^ included data on endometrial hyperplasia/tumor (3621 patients). Analysis of these studies (Fig. 12) showed an increased risk of endometrial adverse events in patients treated with fezolinetant; however, results were statistically insignificant (RR, 1.93; 95% CI, 0.56–6.67; *P* = .30; I2 = 0%).

Similar results were seen in patients treated with 45 mg QD (RR, 3.00; 95% CI, 0.61–14.83) while patients treated with 30 mg QD showed no difference between either treatment (RR, 1.00; 95% CI, 0.14–7.06).

#### 3.5.6. Uterine bleeding.

**Figure 13.**
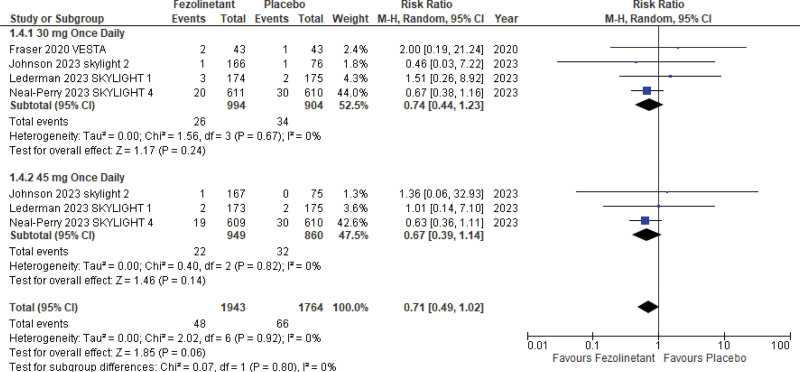
Forest plot for uterine bleeding.

Uterine bleeding was examined in 4 studies^[11,13–15]^ (3707 patients). On pooled analysis, treatment with fezolinetant reported a lesser risk of uterine bleeding over placebo, but the results were not statistically significant (RR, 0.71; 95% CI, 0.49–1.02; *P* = .06; I2 = 0%) (Fig. 13).

In an analysis stratified by different doses, similar effects were observed with treatment with 30 mg QD (RR, 0.74; 95% CI, 0.44–1.23) as well as 45 mg QD dose (RR, 0.67; 95% CI, 0.39–1.14).

## 4. Discussion

NK-3 receptor antagonists are at the forefront of novel treatments for VMS in postmenopausal women. Traditional menopausal hormonal therapy (MHT) has been the most efficacious management, but it comes with a spectrum of side effects and contraindications.^[8,22]^ The completion of ongoing trials for using fezolinetant, an NK-3 receptor antagonist, has shown remarkable results and brought a new advancement in treating VMS. Previous literature on the effectiveness of fezolinetant for VMS lacks statistical analysis.^[23]^ Hence, a pioneering meta-analysis was performed to pool the effect sizes and assess the efficacy and safety of the drug.

The efficacy outcomes in the meta-analysis include the improvement in terms of VMS frequency and severity scores along with MENQoL, HFRDIS, and GCS scores. The data were stratified according to dosage (30 and 45mg QD) and length of treatment (4 and 12 weeks) for VMS outcomes and MENQoL. The fezolinetant group showed a significant reduction in VMS frequency and severity in all stratifications and an improved MENQoL score at 4 and 12 weeks. It also showed an improved HFRDIS score and GCS score at 90mg doses compared to the control. This newer class of drugs has been shown to modulate numerous neural pathways that include NKB neuropeptide. The offset of balance between inhibitory (estrogen) and stimulatory (NKB) signals to the hypothalamic thermoregulatory centers is brought to equilibrium as soon as fezolinetant antagonizes NKB, resulting in VMS relief.^[12]^ The normalization of KNDy neurons is the probable reason for the effectiveness of fezolinetant, and our statistical analysis establishes a very clear advantage of this intervention over control.

HT, also called menopause hormonal therapy (MHT), has been the conventional treatment for managing VMS at menopause. It includes estrogen alone or in combination with progestogen, given in continuous or cyclic dosage regimens.^[24]^ In a previous study, a 77% reduction in VMS frequency (95% CI, 58.2–87.5) has been reported with oral estrogen.^[25]^ If it is assumed that the baseline rate VMS frequency is comparable in that review and our meta-analysis, we can see that there is a substantially greater reduction from baseline in VMS frequency with MHT than with fezolinetant at 3 months. Similarly, another scientific review reports the efficacy of 2 estrogen therapies, conjugated equine estrogen, and 17-estradiol against placebo,^[26]^ which is significantly higher compared to what we found for fezolinetant in our study. This further establishes the significant efficacy MHT has for reducing VMS in its own capacity, as does fezolinetant. However, safety concerns and the benefit-to-risk balance should be the parameters to consider when declaring the superiority of either therapy over the other.

MHT has been shown to have a very poor safety profile. It is crucial to note that long-term use of HT carries risks, with side effects including but not limited to venous thromboembolism, breast cancer, stroke, and pulmonary embolism.^[8,22]^ A very large-scale RCT enrolling more than 16,000 postmenopausal women receiving estrogen and progesterone against placebo showed increased hazard ratio (HR) for coronary events and breast cancer across ethnic and age groups. The HR was not influenced by prior risk or disease status. The reduction of events of hip fractures and incidence of colorectal carcinoma was not enough to counterbalance the associated risk, hence a rather low benefit-to-risk ratio.^[22]^

In contrast to MHT, fezolinetant seems to present a relatively better safety profile in addressing VMS. The analysis showed “any adverse events” to be comparable across both fezolinetant and control, though study-related TEAEs had a higher risk in the fezolinetant group. Nonetheless, the dropout rates were shown to be pretty uniform across both groups, allowing us to infer that the study-related TEAEs were not significant enough to cause increased discontinuation in the fezolinetant group. This comes in contrast to MHT, for which a meta-analysis showed a significantly increased risk of the occurrence of any adverse events (odds ratio, 1.41; 95% CI, 1.00–1.99; *P* = .05) while a small, statistically insignificant increased risk of dropouts due to adverse events in the MHT group compared to a placebo.^[24]^ Endometrial safety has been a concern of MHT. However, since the included trials reported on this outcome, and endometrial safety has been a major concern when it comes to the already approved menopausal therapies, the data was pooled to see if it had any detrimental effect (due to any unknown mechanism) or if it offered any protection against the adverse effect. While endometrial hyperplasia/tumors demonstrated an increased trend, uterine bleeding showed decreased incidence in the fezolinetant group. Since the number of participants with endometrial hyperplasia and tumors was reported together in most trials, we could not assess the occurrence of endometrial tumors separately in the 2 groups. This is a crucial point for further trials to consider in order to help establish the safety profile of fezolinetant better. Similar concerns have been raised recently in the literature.^[27]^ A slightly increasing trend of hepatotoxicity was also seen in fezolinetant group. However, none of the safety outcome results were statistically significant. This discussion helps to further draw out the superiority of fezolinetant over MHT, not only in long-term health hazards but also in short-term adverse events.

Similar studies can be found on other nonhormonal interventions for treating VMS. One of the factors involved in the modulation of VMS includes the neurotransmitter norepinephrine and its offsetting effect on the thermoregulatory center. This makes it a target for VMS treatment through serotonin-norepinephrine reuptake inhibitors (SNRIs) that reduce norepinephrine release and improve hot flashes.^[28]^ Similarly, γ-aminobutyric acid analogs such as pregabalin and gabapentin are known to reduce adrenergic activity and VMS.^[29]^ The effectiveness of these alternate nonhormonal VMS therapies has been reported in literature,^[28–34]^ having a reasonable benefit-to-risk ratio compared to MHT, putting these at par with our findings of fezolinetant. A systematic review comparing NK-3 antagonists with SNRIs reported the former to have a more effective reduction in hot flashes and also concluded it to be better in terms of safety, with less adverse effects and discontinuations.^[28]^ Such comparisons are scarce in the literature as of now. More vigorous comparisons and head-on trials between fezolinetant and these nonhormonal treatments will provide evidence as to which of these should form the treatment of choice in postmenopausal women, especially those with contraindications to MHT. Breast cancer survivors should be paid more concern in this regard as treatment of hot flushes is quite challenging due to MHT contraindication.^[35,36]^ Several reviews have been conducted to see the efficacy of nonhormonal treatments in such population.^[35,36]^ However, fezolinetant cannot be commented on for this indication due to lack of clinical data, and thus, this forms an important question to be investigated in the future.

## 5. Limitations and future implications

The authors acknowledge the presence of heterogeneity in the data for which the random-effects model of statistics was applied. Also, the follow-up time that was common among all the studies was short, that is, 12 weeks or 3 months. The sample size was small in most of the included studies. Additionally, the analysis did not compare the results of all dosage subgroups reported; stratification could be performed to include only 3 doses of fezolinetant, 30 mg QD, 45 mg QD, and 90 mg BID in some outcomes. There was also a lack of significant results when comparing outcomes pertaining to safety profile. Furthermore, the data related to MENQoL, HFRDIS, and GCS are liable to have a certain degree of subjectivity bias because these scores are based on questionnaires built upon the patient’s reporting of symptoms. Also, a funnel plot for publication bias could not be plotted since the total number of studies included was <10.

The safety outcomes, especially those related to endometrial outcomes, need to be interpreted with caution due to differences in the trial designs (Skylight 1,^[13]^ 2^[14]^ vs Skylight 4,^[15]^ which is primarily concerned with safety evaluation) and different treatment end points (12 weeks^[13,14]^ vs 52 weeks,^[15]^ respectively). The imbalance of endometrial findings across the fezolinetant and placebo group underscores the need for looking in deeper, to assess if there are any risk factors that should be taken into consideration and to come up with suitable strategies to minimize any such predisposition. It is recommended that adverse effects should be studied in trials and observational studies having greater sample sizes, powered for safety outcomes such as hepatotoxicity and other potential AEs of interest, enrolling populations of various indications, and following up over longer periods of time.

## 6. Conclusions

The results of this study bring to light the effectiveness of fezolinetant as a treatment option, especially for patients at risk of breast cancer and other contraindications of HT. Furthermore, a statistically insignificant (*P* > .05) increase in the risk of endometrial hyperplasia or tumors warrants the need for further research and more exclusive data to establish superiority over conventional estrogen therapy. Finally, it is imperative that the safety profile of fezolinetant is explored exclusively in further trials to establish more credible evidence for inclusion into menopausal therapy guidelines.

## Author contributions

**Conceptualization:** Ummi Aiman Rahman, Talha Bin Kashif, Muhammad Hasanain, Huzaifa Ahmad Cheema.

**Data curation:** Ummi Aiman Rahman, Talha Bin Kashif, Muhammad Usman, Maham Rana, Muhammad Hasanain, Huzaifa Ahmad Cheema.

**Methodology:** Ummi Aiman Rahman, Talha Bin Kashif, Muhammad Hasanain, Muhammad Umair Anjum, Huzaifa Ahmad Cheema.

**Resources:** Ummi Aiman Rahman, Talha Bin Kashif, Huda Jaffar, Pratik Bhattarai.

**Writing—original draft:** Ummi Aiman Rahman, Talha Bin Kashif, Muhammad Usman, Maham Rana, Muhammad Hasanain, Huda Jaffar, Pratik Bhattarai.

**Writing—review & editing:** Ummi Aiman Rahman, Muhammad Hasanain, Muhammad Umair Anjum, Huzaifa Ahmad Cheema, Huda Jaffar.

**Formal analysis:** Muhammad Usman, Maham Rana, Muhammad Umair Anjum.

**Investigation:** Muhammad Hasanain, Huzaifa Ahmad Cheema, Huda Jaffar, Pratik Bhattarai.

**Validation:** Muhammad Umair Anjum, Huzaifa Ahmad Cheema.

## Supplementary Material


