# Supplementary material for: Efficacy and safety of fezolinetant, a neurokinin-3 antagonist, in treating vasomotor symptoms in postmenopausal women: A systematic review and meta-analysis
Source: Medicine (Baltimore). 2023 Dec 15;102(50):e36592. doi: 10.1097/MD.0000000000036592 (PMC10727556; doi:10.1097/MD.0000000000036592)
Supplement: Supplementary file 1 [file medi-102-e36592-s001.docx]

**Supplementary Table S1: Detailed search strategy**

| Database | Search strategy | Results |
| --- | --- | --- |
| MEDLINE | (fezolinetant OR ESN364 OR NK3 Receptor Antagonist OR Neurokinin 3 Receptor Antagonist OR Non-hormonal therapy) AND (Menopause OR menopausal Women OR Menopausal Symptoms OR Flashes OR hot flashes OR Vasomotor Symptoms OR VMS) | 382 |
| Google Scholar | (fezolinetant OR ESN364 OR NK3 Receptor Antagonist OR Neurokinin 3 Receptor Antagonist OR Non-hormonal therapy) AND (Menopause OR menopausal Women OR Menopausal Symptoms OR Flashes OR hot flashes OR Vasomotor Symptoms OR VMS) | 205 |
| Cochrane | (fezolinetant OR ESN364 OR NK3 Receptor Antagonist OR Neurokinin 3 Receptor Antagonist OR Non-hormonal therapy) AND (Menopause OR menopausal Women OR Menopausal Symptoms OR Flashes OR hot flashes OR Vasomotor Symptoms OR VMS) | 155 |
